# A Case of Central Diabetes Insipidus Secondary to Neurosarcoidosis

**DOI:** 10.7759/cureus.11119

**Published:** 2020-10-23

**Authors:** Colleen E Johns, Caroline S Johns

**Affiliations:** 1 Medicine, Ross University School of Medicine, Bridgetown, BRB; 2 Endocrinology, University of South Florida Morsani College of Medicine, Tampa, USA

**Keywords:** central diabetes insipidus, neurosarcoidosis

## Abstract

We present a case of central diabetes insipidus (DI) secondary to neurosarcoidosis. The path to final diagnosis was challenging. Along with reporting the case, we review the available medical literature relating to neurosarcoidosis and central diabetes insipidus in this case report. Patient is a 56-year-old female with notable history of rheumatoid arthritis, anxiety, asthma, hypertension, spinal stenosis, and seizures of unknown etiology who presented to the emergency department for worsening headache for one week. She also endorsed decreased vision, photophobia, nausea, vomiting, gait abnormality, polyuria, and polydipsia over the past three months. Physical exam and neurological exam were unremarkable. Labs on presentation were notable for hypernatremia, increased serum osmolality and urine output of 5 L/day. Given her persistent headache and history of seizure, she underwent a CT head without contrast which showed a posterior suprasellar soft tissue fullness measuring 6 mm in the hypothalamic region. She then had additional imaging studies of the brain including MRI brain w/wo contrast and MRI pituitary w/wo contrast. MRI of the brain showed enlarged optic chiasm with increased T2 signal involving the proximal optic nerves and bilateral optic tracts, no brain parenchymal lesions to suggest multiple sclerosis. MRI of the pituitary w/wo contrast showed suprasellar mass arising from either the hypothalamus or less likely the chiasm which was concerning for high-grade glioma initially. Lumbar puncture was done that showed lymphocytic pleocytosis. Patient underwent right supraorbital craniotomy for biopsy of the suprasellar lesion. Surgical pathology showed noncaseating granulomatous inflammation most consistent with neuro-sarcoidosis. The diagnosis of neurosarcoidosis was made and patient was started on high dose steroids. Although central DI can be seen as a post-op complication, in our case, based on the clinical presentation, labs and imaging, there was concern of central DI on initial presentation. Patient was started on desmopressin 50 mg twice a day which resulted in marked improvement in urine output, serum sodium, and osmolality. Although it is rare to have nervous system involvement of sarcoidosis, symptoms of central diabetes insipidus could represent the first clinical manifestations of neurosarcoidosis. Proper treatment should be initiated in a timely fashion to avoid complications such as hydrocephalus.

## Introduction

Sarcoidosis is described by an immune-mediated process with the appearance of non-caseating granulomas most commonly affecting the respiratory system, lymphatic system, and heart. It is rare with an incidence of 0.2/100,000 to have sarcoidosis affect the central nervous system. About 5-15% of patients with sarcoidosis have central nervous system (CNS) involvement [1]. All types of sarcoidosis including neurosarcoidosis have a mortality rate of 1-5% and deaths are caused mainly by respiratory, cardiac, and neurologic disease [2].

Neurosarcoidosis results in a dysregulation of the hypothalamus-pituitary axis due to the granulomatous infiltrative process [1]. Available literature reveals that infiltration of the posterior pituitary is most likely connected to the clinical manifestations of central diabetes insipidus. Vasopressin, also known as antidiuretic hormone (ADH), is a hormone secreted by the posterior pituitary. The primary function of antidiuretic hormone is to regulate water balance in the body, thirst, and kidney function. Antidiuretic hormone plays a role in increasing the reabsorption of water back into the circulation. Due to the damage in the posterior pituitary caused by neurosarcoidosis, there is a deficiency of antidiuretic hormone. This leads to an increase in the amount of water passed out in the urine. Electrolyte disturbances can occur due to the dysregulation of water balance. Thus, patients present with polyuria (urine output more than 3 L/day in adults) and polydipsia [3].

The primary treatment for central diabetes insipidus is desmopressin. Desmopressin is an ADH analog and it functions similarly to ADH. The different routes of desmopressin administration include oral, intranasal, subcutaneous, and intravenous. As patients consume too much water from excessive thirst, hyponatremia can occur. This can lead to brain injury as cells begin to swell. Therefore, it is critical to monitor for hyponatremia in patients with central diabetes insipidus [3]. Here, we present a case report with central diabetes insipidus as an initial presenting symptom of neurosarcoidosis in a patient who was not previously diagnosed with sarcoidosis.

## Case presentation

Patient is a 56-year-old female with notable history of rheumatoid arthritis, anxiety, asthma, hypertension, spinal stenosis, and seizures who presented to the emergency department for worsening headache over the past week. She also endorsed decreased vision, photophobia, nausea, vomiting, gait abnormality, polyuria, and polydipsia over the last few months. On physical examination, the patient was awake and alert. On presentation, her pulse rate was 74, blood pressure 130/85 and was afebrile. Physical examination was otherwise unremarkable. Neurological examination revealed unremarkable motor and sensory examination.

**Figure 1:**
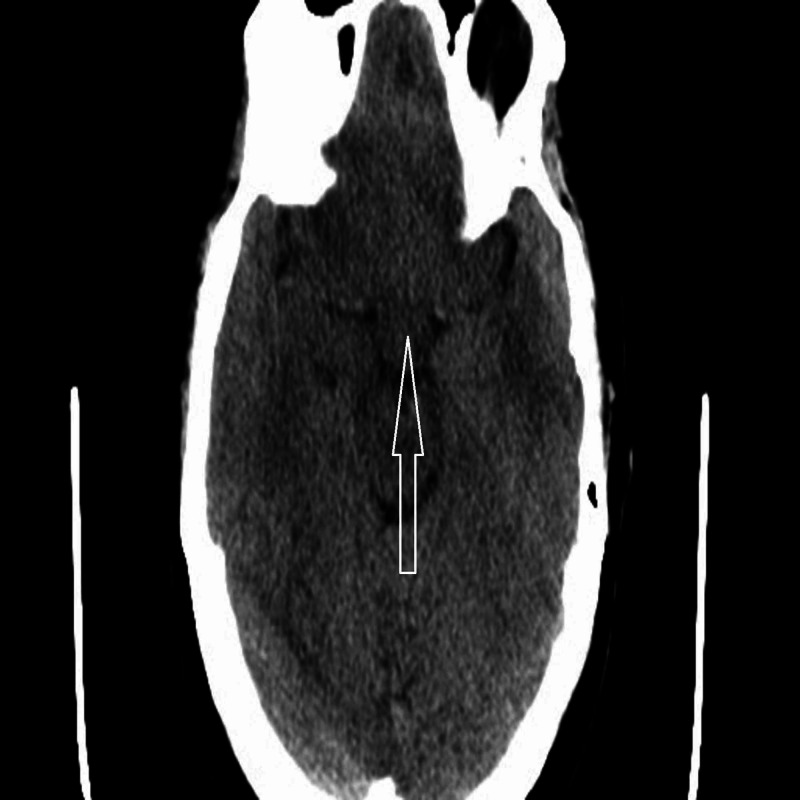
CT of the head without contrast showing a posterior suprasellar soft tissue fullness measuring 6mm in the hypothalamic region.

Laboratory investigations on presentation included complete blood count with white blood cells 5.12, hemoglobin 12.0, hematocrit 36.7, and platelets 173. Basic metabolic panel was remarkable for sodium 157, potassium 4.9, chloride 114, CO_2_ 21, blood urea nitrogen 4, creatinine 0.8. Liver function tests were total bilirubin 0.6, alkaline phosphate 62, alanine aminotransferase 32, and aspartate aminotransferase 37. Given hypernatremia, additional labs were obtained including blood osmolality 334 MOS/Kg, urine osmolality 179 MOSM/kg and urine Na 45 MEQ/L. Given her persistent headache and history of seizure, she underwent a CT head without contrast which showed a posterior suprasellar soft tissue fullness measuring 6 mm in the hypothalamic region (Figure 1). 

**Figure 2:**
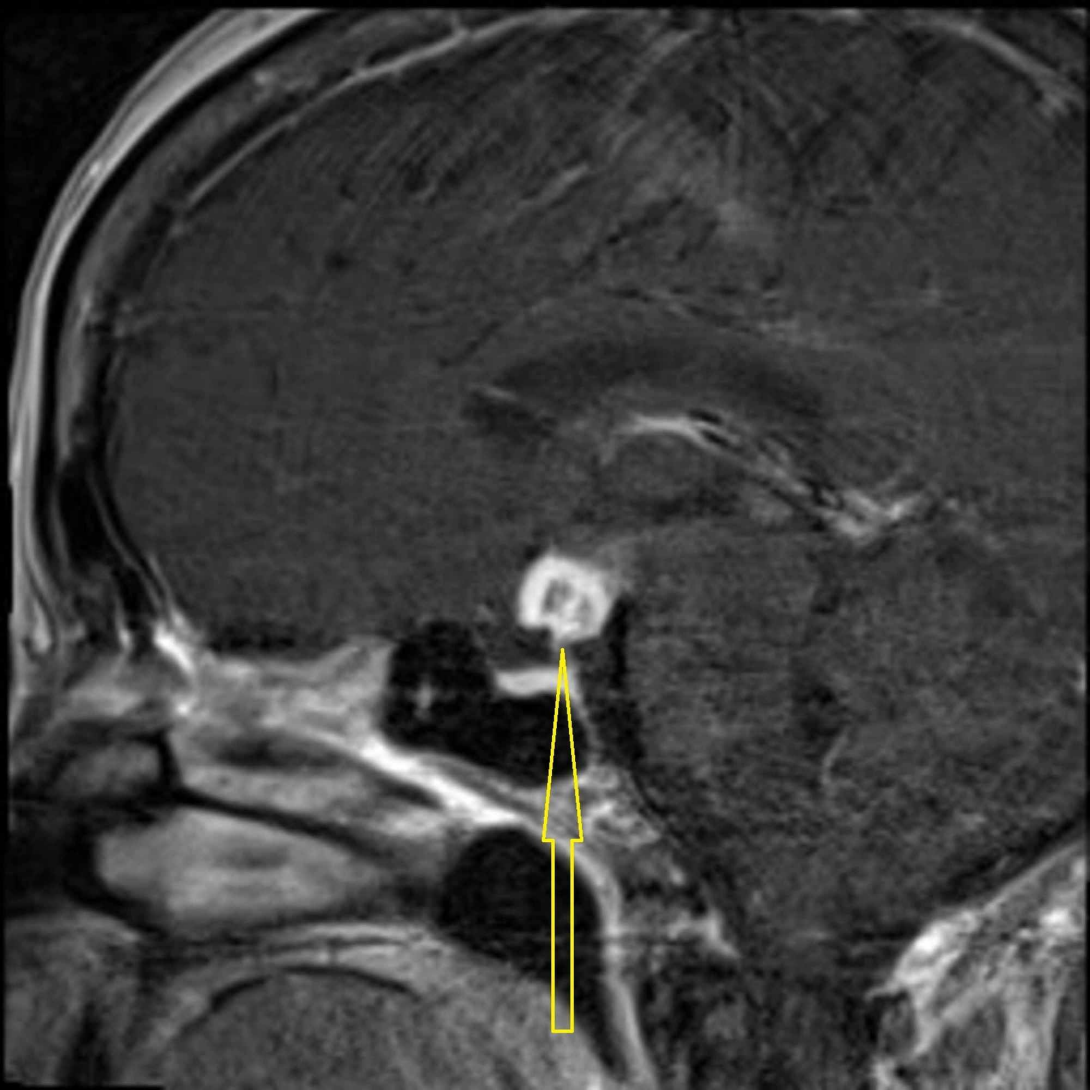
MRI of the pituitary w/wo contrast showed suprasellar mass arising from either the hypothalamus or less likely the chiasm.

She then had additional imaging studies of the brain including MRI brain w/wo contrast and MRI pituitary w/wo contrast. MRI of the brain showed enlarged optic chiasm with increased T2 signal involving the proximal optic nerves and bilateral optic tracts and no brain parenchymal lesions to suggest multiple sclerosis. MRI of the pituitary w/wo contrast showed suprasellar mass arising from either the hypothalamus or less likely the chiasm which was concerning for high-grade glioma initially. There was no leptomeningeal enhancement noted and the pituitary and the stalk appeared normal (Figure 2). 

During hospitalization, patient underwent a lumbar puncture (LP) with studies demonstrating xanthochromia, 482 nucleated cells with 29% neutrophils and 60% lymphocytes, 12 red blood cells, glucose 41 and total protein 271, most consistent with lymphocytic pleocytosis. She was started on broad-spectrum antibiotics (cefepime and vancomycin) initially. Routine electroencephalogram (EEG) was negative for seizures. Ophthalmology was consulted based on the MRI findings and patient was found to have normal visual acuity, visual fields consistent with left superior homonymous quadrantanopia, with no papilledema. Neurosurgery was also consulted and after discussion with the tumor board, plan was to obtain biopsy of the lesion. Patient underwent right supraorbital craniotomy for biopsy of the lesion. Surgical pathology showed noncaseating granulomatous inflammation most consistent with neuro-sarcoidosis. Her post-op labs were remarkable for cortisol <1.0, indicating adrenal insufficiency. No other endocrinopathies were noted pre- and post-surgery. Her serum and CNS angiotensin-converting enzyme (ACE) were negative. Other tests including cerebrospinal fluid (CSF) cytology, bacterial CSF culture, meningitis panel, HIV, and tuberculosis TB-Quantiferon test were all negative. Patient also had CT chest as part of sarcoidosis workup. CT chest showed no hilar or mediastinal adenopathy concerning for sarcoidosis. 

When the diagnosis of central diabetes insipidus and neurosarcoidosis was initially made during hospitalization, patient was started on high-dose steroids for five days together with desmopressin 50 mg twice a day. She was discharged home and advised to continue follow-up as an outpatient. Patient continued to follow up with multi-specialty teams including neurosurgery, rheumatology, endocrinology, neurology and ophthalmology. She continued to be on desmopressin intranasal 10 mcg twice a day for central diabetes insipidus and as her post-op labs suggested secondary adrenal insufficiency, she continued to be on hydrocortisone 30 mg daily. During her follow-up visit with rheumatology, patient refused to continue prednisone due to side effects, therefore she was started on CellCept and infliximab for further treatment of her neurosarcoidosis.

## Discussion

Sarcoidosis is a disease that can have varying clinical presentation. Hypothalamus, pituitary and cranial nerves are the most frequently affected regions of the nervous system. The differential diagnosis that must be taken into consideration should include infectious diseases, autoimmune inflammatory diseases and tumors such as lymphoma, meningioma carcinomatosis, and gliomas. Available data suggests that genetic, individual and environmental factors play a role in the etiology of neurosarcoidosis. Sarcoid tissues have shown the presence of protein antigens and mycobacterial DNA which indicates that the establishment of neurosarcoidosis may have microbial involvement [4].

Isolated neurosarcoidosis can present with headache in 90% of the cases followed by paresthesia in 50% of the cases whereas the neurological manifestations of systemic sarcoidosis include peripheral neuropathy presenting in 69% of the cases and headache showing in only 48% of the cases. Neuropsychiatric features such as dementia, depression, psychosis, poor concentration and hallucinations can be found in some cases of patients with neurosarcoidosis (Table 1) [4]. 

**Table 1:** Clinical manifestations of neurosarcoidosis

1	Headache
2	Peripheral neuropathy
3	Paresthesia
4	Dementia
5	Depression
6	Psychosis
7	Poor concentration
8	Hallucinations

Studies show that infiltration of the posterior pituitary is highly likely to be associated with clinical presentations of central diabetes insipidus. As discussed earlier, patients present with polyuria and polydipsia. The usual findings seen on an MRI include an expansion of the pituitary and a thicker pituitary stalk. Patient may need lifelong desmopressin therapy as the recovery of normal endocrine function is less likely to occur. The presence of neurological disease in sarcoidosis is considered as an absolute indication to begin treatment immediately [5]. Some patients can develop granulomas on the fourth ventricle leading to hydrocephalus demanding emergency treatment [4].

Patients with presumed neurosarcoidosis should get an MRI of the brain. Diagnosis must be made based on clinical and radiologic findings and must be confirmed with a biopsy [4]. Diagnostic imaging modality for neurosarcoidosis is gadolinium-enhanced MRI [5]. The anomalies identified in an MRI include periventricular white matter lesions, meningitis/meningoencephalitis, solid parenchymal enhancing lesions, cranial neuritis, and myelopathy. Primary treatment includes glucocorticoids, usually prednisone [6]. In patients who cannot tolerate steroid monotherapy due to its side effects or in patients whose symptoms are severe, second-line treatments include immunosuppressive agents such as methotrexate, cyclophosphamide, infliximab, and hydroxychloroquine [1,2,6]. In cases where medical therapy fails, radiotherapy can be used [4].

## Conclusions

Although it is rare to have nervous system involvement of sarcoidosis, the initial presenting symptoms of central diabetes insipidus could represent the first clinical manifestation of neurosarcoidosis. When patients present with symptoms of central diabetes insipidus such as polyuria and polydipsia initially, it is important to investigate further by performing an MRI of the brain to look for the presence of neurosarcoidosis. The key learning points on neurosarcoidosis and central diabetes insipidus include the following. Sarcoidosis is described by an immune-mediated process with the appearance of non-caseating granulomas in the respiratory system, lymphatic system, and heart. Neurosarcoidosis results in a dysregulation of the hypothalamus-pituitary axis due to the granulomatous infiltrative process. This leads to central diabetes insipidus and patients present with polyuria and polydipsia. Diagnostic imaging modality for neurosarcoidosis is gadolinium-enhanced MRI. In some patients, neurosarcoidosis can lead to complications such as hydrocephalus due to the development of granulomas on the fourth ventricle. Therefore, therapy should be initiated immediately. Primary treatment for central diabetes insipidus is administration of desmopressin and primary treatment for neurosarcoidosis includes glucocorticoids usually prednisone. In patients who cannot tolerate steroid monotherapy, immunosuppressive agents are used.
